# Prevalence and Risk Factors of Dry Eye Disease in the South of Palestine

**DOI:** 10.21315/mjms2024.31.2.8

**Published:** 2024-04-23

**Authors:** Mohammed Aljarousha, Noor Ezailina Badarudin, Mohd Zulfaezal CHE Azemin, Yousef Aljeesh, Abuimara Amer, Muhammad Afzam Shah Abdul rahim

**Affiliations:** 1 Department of Optometry and Visual Science, Kulliyyah of Allied Health Sciences, International Islamic University Malaysia, Pahang, Malaysia; 2 Department of Optometry, Faculty of Health Science, Islamic University of Gaza, Gaza Strip, Palestine; 3 College of Nursing, Islamic University of Gaza, Gaza Strip, Palestine; 4 European Gaza Hospital, Department of Optometry, Gaza Strip, Palestine

**Keywords:** dry eye disease, OSDI, aqueous tear deficiency, evaporative dry eye, clinical signs

## Abstract

**Background:**

The prevalence of dry eye disease (DED) is rising globally and needs to be urgently addressed by medical professionals because it lowers patients’ quality of life. There are as yet no available data in the literature about the prevalence of and risk factors for DED in the Gaza Strip, a gap that the present study seeks to address.

**Methods:**

A cross-sectional study was carried out between March and August 2022 in Gaza governorates using a proportional stratified sampling technique. Only Gazan individuals ≥ 18 years old and able to follow the instructions were included. The Ocular Surface Disease Index (OSDI) questionnaire, which has previously been translated into Arabic and validated, was applied to evaluate DED symptoms. Subjective clinical tests for DED conducted were tear meniscus height (TMH), meibomian gland dysfunctions (MGDs), Marx line (ML), conjunctival Lissamine green staining (LGS), tear film break-up time test (TBUT), corneal fluorescein staining (CFS) and Schirmer II tear test (STT). DED was defined based on an Arab-OSDI score ≥ 13 and at least one positive clinical sign.

**Results:**

A total of 426 participants were assessed from four areas (North Gaza Strip, 82; Gaza City, 147; Mid-Zone Gaza Strip, 62; South Gaza Strip, 135). The prevalence of DED in the present study was 31.5% (95% CI: 27.1, 36.1). Age > 50 years old (odds ratio [OR] = 10.45; 95% CI: 2.95, 37.05; *P* < 0.001), female gender (OR = 3.24; 95% CI: 1.40, 7.52, *P* = 0.006), menopause or pregnancy (OR = 2.59; 95% CI: 1.25, 5.35; *P* = 0.03) and pharmacotherapy (artificial tears; OR = 9.91; 95% CI: 2.77, 35.46; *P* < 0.001) were each associated with DED symptoms. South Gaza Strip (OR = 0.04; 95% CI: 0.01, 0.12; *P* < 0.001), unemployed (OR = 11.67; 95% CI: 1.43, 95.44; *P* = 0.02), non-consumption of caffeine (OR = 0.40; 95% CI: 0.19, 0.88; *P* = 0.02) and TMH < 0.2 (OR = 1.80; 95% CI: 1.02, 3.19; *P* = 0.04) were associated with TBUT < 5 s. LGS was associated with those > 50 years old (OR = 2.70; 95% CI: 1.38, 5.28; *P* = 0.004), previous refractive or ocular surface surgeries (OR = 2.97; 95% CI: 1.34, 6.59; *P* = 0.008) and CFS ≥ 1 (OR = 1.91; 95% CI: 1.07, 3.44; *P* = 0.03).

**Conclusion:**

Various aspects of DED were linked with different risk factors, suggesting that DED subtypes have different underlying pathophysiologies.

## Introduction

Dry eye disease (DED) is a serious public health issue since it impairs vision-related quality of life, especially in individuals with moderate or severe DED (1, 2). The experts attending the Tear Film and Ocular Surface Society Dry Eye Workshop II (TFOS DEWS II) in 2017 refined the definition of DED as ‘a multifactorial interpalpebral ocular surface pathology and problems in the tear film profile that may contribute to a variety of ocular symptoms, fatigue, discomfort and fluctuation of vision’ (1). In addition, instability and hyperosmolarity of the tear film, inflammation and damage on the ocular surface and neurosensory abnormalities play aetiological roles (3). The causes of DED include inadequate aqueous tear secretion, increased tear evaporation, meibomian gland dysfunctions (MGDs) and/or disruption of the corneal epithelium (4). The estimated prevalence of DED ranges from 20% to 50% around the globe (5). The prevalence rate varies so widely in studies because of dissimilarities in the criteria applied for diagnosis, age range, specific regions and types of study populations used (6, 7). Cross-sectional studies have noted that the pathology is more common among females, those of advanced age, contact lens users, those with previous refractive or ocular surface surgeries and smokers (8–11). Systemic medical conditions such as diabetes mellitus and hypertension, systemic use of medications such as aspirin and antihistamines, history of ocular disorders such as blepharitis, ocular allergy, glaucoma and pterygium, vitamin A or D deficiency, shortage of sleep and MGDs are other potential risk factors found to impact DED prevalence (12–16). Epidemiological studies have revealed poor correlations between the questionnaires on DED symptoms and clinical signs (17, 18). Therefore, DED should be assessed based on symptoms in conjunction with signs. Previous studies employed validated questionnaires such as the Ocular Surface Disease Index (OSDI) (19, 20). In the present study, the Arab-OSDI was used to quantitatively evaluate the prevalence and risk factors of symptomatic DED in the population of the Gaza Strip. The Arab-OSDI version is a highly effective and reliable measure for evaluating quality of life, ocular discomfort and dry eye symptoms. Moreover, it can be applied consistently and repeatedly, ensuring accurate and consistent results (21). Complementing this assessment, various clinical evaluations such as the Schirmer II tear test (STT), tear film break-up time test (TBUT), conjunctival Lissamine green staining (LGS), corneal fluorescein staining (CFS), tear meniscus height (TMH), Marx line (ML) and MGD assessment can be conducted. These additional measures enhance the comprehensive evaluation of ocular health and provide valuable insights into various aspects of eye conditions (19–22). To our knowledge, there are no available data on the prevalence and risk factors of DED in the Gaza Strip. Therefore, this study aimed to estimate DED’s prevalence and risk factors for the area’s population.

## Methods

The report and presentation for this cross-sectional study are in compliance with STROBE (Strengthening the Reporting of Observational Studies in Epidemiology) guidelines. The report’s title and abstract, introduction, methods, findings, commentary and other material all accord with the recommendations in the checklist (Appendix).

### Study Design and Sample

This study was conducted in four areas: North Gaza Strip, Gaza City, Mid-Zone Gaza Strip and South Gaza Strip. Proportional stratified sampling was used to calculate the sample size required, and data were collected between March and August 2022. Based on the calculations, the minimum sample size required was approximately 384, as shown in the formula *n* = *p* (1–*p*) *Z*^2^_1– α/2_/d^2^ (23). This value was derived using a *Z* = 1.96 for a CI of 95%, *p* = 50% and *d* = 5%. However, based on Yasir et al. (16), the sample size was increased by 10% (amounting to 426 participants) to compensate for any data loss and to increase the study’s representativeness and generalisability. Based on the total population in each area, the number of participants (volunteers) in each area were as follows: 135 in South Gaza Strip, 62 in Mid-Zone Gaza Strip, 147 in Gaza City and 82 in North Gaza Strip. Participants were recruited at random; there were four strata, each of which received a proportional allocation. Data regarding risk factors were obtained by asking participants directly and confirmed with Palestinian Ministry of Health online records (8, 16), as listed in Table 1.

### Inclusion and Exclusion Criteria

Only Gazans who were at least 18 years old and could follow instructions were included. Key exclusion criteria included individuals with positive ocular surface disorders such as inflammation and those with a history of ocular surface or refractive surgery within the previous 12 months.

### Subjective Symptoms of Dry Eye Disease

The Arab-OSDI questionnaire has been applied to evaluate DED-related symptoms. It consists of 12 questions focusing on ocular symptoms and vision-related and environmental triggers, which have been translated into Arabic and subsequently validated (21). The Arab-OSDI grades are normal = 0–12, mild = 13–22, moderate =23–32 and severe = 33–100. Participants with Arab-OSDI grades of > 13 were considered positive for DED (18, 24).

### Examinations

The objective clinical tests for DED in this study involved TMH, MGDs, ML, LGS, TBUT, CFS and STT. TMH was examined by minimising the shape of a slit lamp beam and arranging the beam horizontally in alignment with the lower eyelid rim. A participant with TMH < 0.2 mm was diagnosed as having inadequate aqueous tear production (25). The obstruction of the meibomian gland was assessed by gently inspecting the eyelid margin with slit lamp biomicroscopy. Grading of MGDs ranged from 0 point to 4 points (Grade 0 exhibits clear meibum, Grade 1 exhibits coloured meibum with normal consistency, Grade 2 exhibits viscous meibum, Grade 3 exhibits inspissated meibum and Grade 4 exhibits a blocked meibomian gland) (26). The measurement of ML was carried out by moistening a LGS strip with non-preserved saline solution and soaking it in the lower fornix. The lid margin area was divided into three zones (inner, middle and outer) and each region was graded on a scale from 0 to 3: a line entirely on the conjunctival side of the meibomian orifices is Grade 0, a line that touches the orifices in any way is Grade 1, a line that passes through every orifice is Grade 2, and a line that is on the eyelid margin side of the orifices are represented by Grade 3. An ML score of > 3.5 was considered abnormal (27). The conjunctiva was also assessed for the level of LGS. Grading of conjunctival surface staining ranged from 0 to 5 (Grade 0 denotes an absence of the conjunctival surface staining, Grade 1 denotes minimal staining limited to 10 dots of the conjunctiva, Grade 2 denotes mild staining of 32 dots of the conjunctiva, Grade 3 denotes moderate staining of 100 dots of the conjunctiva, Grade 4 denotes marked staining of 316 dots of the conjunctiva and Grade 5 denotes severe staining of > 316 dots of the conjunctiva) (28). TBUT was assessed by applying a fluorescein strip with a cobalt blue slit lamp beam in participants with DED. A dry fluorescein strip moistened with a single drop of saline was placed in contact with the bulbar conjunctiva. The period from the last blink to the appearance of random dark spots and/or streaks in the tear film was recorded as TBUT. Three readings were taken consecutively and averaged for each eye as the actual value in seconds (29). The dye was subsequently used to detect invasive staining on the corneal epithelial barrier, which appeared green when illuminated with cobalt blue light. Redness grading of the CFS was then applied (30), using a scale from 0 to 3 (Grade 0, no staining of the corneal epithelial surface; Grade 1, mild staining confined to no more than one third of the cornea; Grade 2, moderate staining of no more than one half of the cornea; and Grade 3, severe staining of no more than one half of the cornea). STT was performed with local anaesthesia by inserting Schirmer filter paper laterally in the lower fornix. The filter paper was removed after 5 min and the wet part was recorded in in mm. A participant with STT < 15 mm was considered to have a deficiency in aqueous tear secretion (31).

### Diagnostic Criteria of DED

Our research team defined the overall prevalence of DED based on positive symptoms (Arab-OSDI ≥ 13) and at least one positive clinical sign (TMH < 0.2 mm, MGDs > 1, ML > 3.5, LGS ≥ 1, TBUT < 5 s, CFS ≥ 1 or STT < 15 mm), as presented in Table 2. The outcomes of clinical signs in two eyes were considered; in the event of fluctuations between eyes, raw data from the worse eye were used for analysis.

### Statistical Analysis

Data were analysed with IBM SPSS (version 23.0, SPSS Inc., Chicago, Illinois, USA). The Shapiro-Wilk test was used to evaluate the normality of distribution. The prevalence of participants with dry eye, based on symptoms and clinical signs, were described using means, standard deviations and percentages, as appropriate. Categorical variables (risk factors and severity of dry eye symptoms) were analysed using a chi-square test. Bivariate and logistic regression models were used to evaluate the OR of and risk factors for DED. The alpha level was set at *P* < 0.05.

## Results

### Dry Eye Disease Prevalence

**Figure 1:**
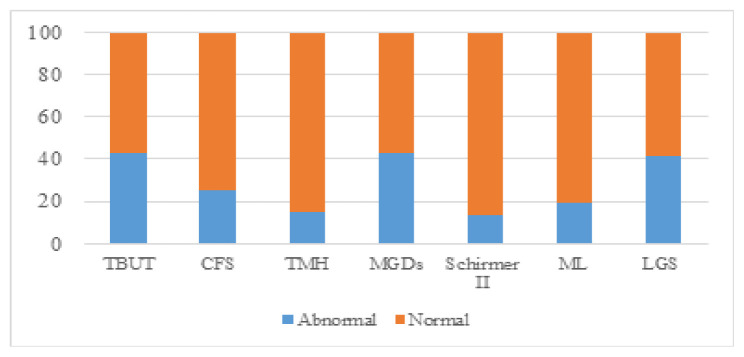
Percentage of participants with/without positive dry eye clinical signs

Approximately 426 participants were recruited from four areas [North Gaza Strip (*n* = 82), Gaza City (*n* = 147), Mid-Zone Gaza Strip (*n* = 62) and South Gaza Strip (*n* = 135)]. In the study population, the mean score for the OSDI (mean ± SD) was 21.10 ± 18.13. The maximum OSDI score on sub-scale (A) for ocular symptoms was 20; the maximum vision-related score for sub-scale (B) was 16 and the maximum environmental triggers score on sub-scale (C) was 12. A total of 248 participants (58.2%; 95% CI: 53.4, 62.9) were diagnosed with dry eye symptoms (Arab-OSDI score ≥ 13), ranging from mild (17.6%; 95% CI: 14.1, 21.6) through moderate (12.0%; 95% CI: 9.0, 15.4) to severe (28.6%; 95% CI: 24.4, 33.2). Based on the definition of at least one positive dry eye clinical sign, the proportion of DED was 95.1% (95% CI: 92.6%, 96.9%). The prevalence of DED was higher in MGDs (43.2%; 95% CI: 38.4, 48.0), followed by TBUT (42.7%; 95% CI: 38.0, 47.6), LGS (38.7%; 95% CI: 34.1, 43.5), CFS (25.1%; 95% CI: 21.1, 29.5), ML (19.7%; 95% CI: 16.0, 23.8), TMH (15.3%; 95% CI: 12.0, 19.0) and the STT (13.4%; 95% CI: 10.3, 17.0) as shown in Table 3. Approximately 134 participants had an Arab-OSDI score ≥ 13 and at least one positive clinical sign. The prevalence of DED in the present study was 31.5% (95% CI: 27.1, 36.1); of that number, 48 participants had aqueous tear deficiency (ATD) but not evaporative dry eye (EDE; 11.3%; 95% CI: 8.4, 14.7), 121 participants had EDE but not ATD (28.4%; 95% CI: 24.2, 32.9) and 42 participants (9.9%; 95% CI: 7.2, 13.1) had mixed DED of both (Figure 1).

### Risk Factor Assessment for Symptomatic DED

**Figure 2:**
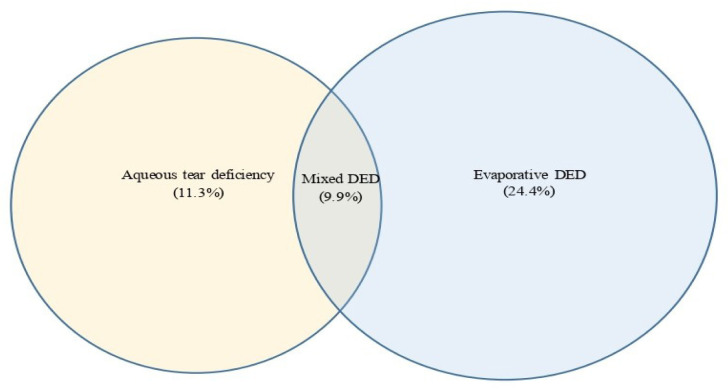
Area-proportional Venn diagram of proportion of participants with evaporative dry eye, aqueous tear deficiency and mixed DED

As to prevalence of Arab-OSDI by gender, females were found to have higher scores (≥ 13) than males (70.7%; 95% CI: 63.7, 77.1 and 48.3%; 95% CI: 41.8, 54.9, respectively) at a statistically significant level (*P* < 0.001). When comparing the four regions, the prevalence of symptomatic DED based on the Arab-OSDI version was highest in the South Gaza Strip (69.6%; 95% CI: 61.1, 77.2) and lowest in the Mid-Zone Gaza Strip (38.7%; 95% CI: 26.6, 51.9). A significant correlation was also observed in symptomatic DED among regions (*P* < 0.001). The prevalence of symptomatic DED was lowest among participants aged 18 years old–30 years old and highest among participants ≥ 50 years old (*P* < 0.001) (Figure 2). The difference in Arab-OSDI score was significant in terms of rotating shift work patterns (*P* = 0.04). The frequency of symptomatic DED was higher in participants with such a pattern (61.2%; 95% CI: 49.7, 71.9) than those who worked a regular day shift (55.1%; 95% CI: 46.0, 63.9). Statistical significance was also noted regarding pharmacotherapy use (i.e. artificial tears; *P* = 0.005), with participants using artificial tears having a higher severity of dry eye symptoms (71.3%; 95% CI: 61.0, 80.1) than those who did not (54.5%; 95% CI: 49.0, 60.0). Furthermore, dry eye symptoms were significantly associated with a vegetarian diet and a history of eye diseases (*P* = 0.01).

A logistic regression model was carried out to identify predictors of Arab-OSDI scores ≥ 13. The significant factors associated with symptomatic DED were older age group (> 50 years old; OR = 10.45; 95% CI: 2.95, 37.05; *P* < 0.001), female gender (OR = 3.24; 95% CI: 1.40, 7.52; *P* = 0.006), menopause or pregnancy (OR = 2.59; 95% CI: 1.25, 5.35; *P* = 0.03) and pharmacotherapy (i.e. artificial tears; OR = 9.91; 95% CI: 2.77, 35.46; *P* < 0.001), as summarised in Table 4.

### Risk Factor Assessment for Clinical Tests

In the adjusted model, participants who lived in the Mid-Zone Gaza Strip and South Gaza Strip were 92% (OR = 0.08; 95% CI: 0.03, 0.26; *P* < 0.001) and 96% (OR = 0.04; 95% CI: 0.01, 0.12; *P* < 0.001) less likely to be diagnosed with TBUT < 5 s than participants who lived in the North Gaza Strip. Oral contraceptive or hormonal therapy use was also associated with higher odds of a TBUT outcome < 5 seconds (OR = 2.88; 95% CI: 1.04, 8.00; *P* = 0.04). The unemployed were 11 times more likely to be diagnosed with TBUT < 5 s (OR = 11.67; 95% CI: 1.43, 95.44; *P* = 0.02) than were retired participants. Moreover, those who did not consume caffeine were 60% less likely to be diagnosed with lower TBUT values (OR = 0.40; 95% CI: 0.19, 0.88; *P* = 0.02) than caffeine users. Compared to the reference category of participants with a TMH ≥ 0.2 mm, those with a TMH < 0.2 mm were twice as likely to have low TBUT values (OR = 1.80; 95% CI: 1.02, 3.19; *P* = 0.04). Participants in the 41 years old–50 years old age group had a five times higher risk of low TMH values than those in the 18 years old–30 years old age cohort (OR = 5.05; 95% CI: 1.15, 22.20; *P* = 0.03). Poorer TMH scores were more common in participants who worked rotating shifts than those with regular day shifts (OR = 2.80; 95% CI: 1.08, 7.31; *P* = 0.04). The present study also found that dietary supplement or multivitamin use was associated with lower odds of an outcome of TMH < 0.2 mm (OR = 0.25; 95% CI: 0.09, 0.66; *P* = 0.005). A potential protective factor against CFS was identified in cases of deficiency of vitamin A or D (OR = 0.14; 95% CI: 0.04, 0.53; *P* = 0.004). This may be due to only 9% (n = 10 of 107) of our Gazan participants having vitamin A or D deficiency with CFS ≥ 1. Lower STT values were associated with participants aged 18 years old–30 years old (OR = 2.51; 95% CI: 1.11, 5.66; *P* = 0.026) than with participants aged > 50 years old. Compared to participants who used screens for > 6 h, those who used screens for 3 h–6 h were 56% less likely to have a lower STT score (OR = 0.44; 95% CI: 0.20, 0.94; P = 0.034). Participants who lived in Gaza City (OR = 4.91; 95% CI: 1.2, 19.92; *P* = 0.026) were more likely to have ML staining than those who lived in North Gaza Strip. In addition, LGS (OR = 4.35; 95% CI: 1.57, 12.08; *P* = 0.005) and caffeine consumption (OR = 4.48; 95% CI: 1.50, 13.38; *P* = 0.007) were the two risks factors identified for positive ML staining. None of the independent variables examined was associated with the presence of MGDs in the multivariate logistic regression model. LGS was independently associated with advancing age for participants > 50 years old compared with those aged 18 years old–30 years old (OR = 2.70; 95% CI: 1.38, 5.28; *P* = 0.004), participants with previous refractive or ocular surface surgeries (OR = 2.97; 95% CI: 1.34, 6.59; *P* = 0.008) and CFS (OR = 1.91; 95% CI: 1.07, 3.44; *P* = 0.03), as illustrated in Table 5.

## Discussion

The prevalence of DED in the Middle East has not been extensively studied. This is the first population-based investigation into the prevalence of and risk factors for DED in the Gazan population. Our Arab-OSDI score, 21.10 ± 18.13 in the study population, was also lower than the earlier findings (18, 24). This could be due to the validated Arab-OSDI questionnaire’s ability to remove the language barrier in gleaning information participants. Alhamyani and colleagues (24) found that the OSDI score mean ± SD was highest in sub-scale (A) (ocular symptoms) and lowest in sub-scale (C) (environmental triggers), which was confirmed in the present study. Half of this study’s participants (*n* = 248, 58.2%) had Arab-OSDI scores ≥ 13. This finding is similar to a non-clinical study of a Jordanian population that reported a prevalence of dry eye symptoms of 59%, although with a different OSDI cut-off applied (19).

In the current study, 95.1% of participants had at least one positive clinical sign of DED, which accords with a previous epidemiological study in Mexico (22). The proportion of participants diagnosed with ATD was 11.3%, while a higher percentage (24.4%) was assessed as having EDE. This conforms with earlier studies that reported ATD to be a less common type of DED (4). Our outcomes revealed that MGDs were the most frequently positive clinical sign among the study population. The high prevalence of MGDs in the present study suggests evaporative cause as the most prominent aetiology of dry eye among the Gazan population. In our study population, the prevalence of DED diagnosed using Arab-OSDI ≥ 13 was higher in females than males, which may be due to the smaller lacrimal gland acini in females (32). Further, higher oestrogen levels in females may have an impact on the tear film’s profile (36). This is consistent with a recent study from Dubai (20), which demonstrated that females had 2.06 times higher OSDI scores than males. The OR in the current study, however, was higher than the Dubai study, where it was 3.24. In the present study, DED symptom outcomes revealed a significant difference between participants aged 18 years old–30 years old and > 50 years old, which is consistent with previous studies (6, 33). In the adjusted model, our data showed that older Gazan participants (aged > 50 years old) had higher Arab-OSDI scores and lower TMH and higher LGS scores than the 18 years old–30 years old age cohort. This may be due to the high percentage of systemic diseases in the older age group, such as diabetes mellitus and hypertension, and systemic medications that may influence tear film stability (34, 35). By contrast, participants aged 18 years old–30 years old had a higher prevalence of poorer STT values than those > 50 years old. This could be because greater aqueous tear production compensates for a failed lipid layer to preserve tear homoeostasis, as previously postulated (36), this would result in a ‘falsely’ higher STT value (37).

The current study reported an association between rotating shift work and the Arab-OSDI score. Rotating shift work increases sensitivity to pain and results in sleep disorders, which can aggravate dry eye symptoms, as confirmed by worse TMH scores (38). The present study found an association between artificial tear use and dry eye symptoms, as reported in previous studies (39, 40). Data from the present study showed that menopause or pregnancy poses a significantly higher risk of developing DED symptoms, which has also been reported in previous studies (41, 42). The greater prevalence of DED identified in our study could be attributed to hormonal changes during menopause or pregnancy (41). In addition, the present study demonstrated that a vegetarian diet was associated with a significantly higher risk of developing DED, as previously reported (39). Vegetarians are at elevated risk of not obtaining adequate vitamin D because it is almost entirely contained in animal sources (43). Further, as in previous studies (16, 44), we found a statistically significant difference among the classifications of mild, moderate and severe Arab-OSDI scores based on previous history of eye diseases.

The present study also found a significant association between unemployed participants and TBUT < 5 s. This may be due to the fact that the proportion of unemployed participants was higher among females than males (45). We also observed a significant association between caffeine consumption and positive signs of DED, which may be due to diminished aqueous tear secretion, possibly due to the anticholinergic effects of caffeine (35, 46). According to the outcomes reported here, lower TBUT values had a significant correlation with poorer TMH scores, which accords with studies by Nguyen et al. (47) and Wei et al. (48). Those who consumed dietary supplements or multivitamins were less likely to be diagnosed with lower TMH values than those who did not. However, Martinez et al. (22) reported an insignificant association between the use of multivitamins and ATD among the Mexican population they studied. This could be due to differences in the diagnostic criteria employed for ATD between the two studies.

A negative correlation was reported between screen time hours and STT values; that is, as screen time hours increased, STT values decreased. According to previous studies, blink rates decrease when focusing on a proximate object from the normal 20 times per min to roughly 10 times per min, which causes dry eye (49, 50). A significant association was noted between positive ML staining and participants from Gaza City. This might be due to the LGS showing ocular surface damage before symptoms and signs of DED appeared (51). LGS was chosen as the ocular dry tool for the ML as it is optimal for detecting dead or deteriorated cells but does not stain healthy cells (52). The multivariate analysis demonstrated that LGS was significantly associated with previous refractive or ocular surface surgeries in our sample population. This could be caused by the phototoxic effect of the reactive oxygen species produced by a light microscope’s operation, which can cause the devitalisation of conjunctival and corneal epithelial cells, squamous metaplasia of the conjunctival epithelium and a decrease in conjunctival goblet cell density (53). The sample population also showed a significant association between higher LGS and higher CFS values. This might be because conjunctival, corneal and ML staining have been found to have a poor ability to detect dry eye in mild to moderate cases (54).

Like any research, the present study has certain limitations. It lacks information on the duration and type of contact lens used and the types of diabetes mellitus (because the numbers of both contact lens users and diabetic participants were small), which may lead to an underestimation of the actual prevalence of DED in the current study. The inclusion of participants from Gaza City was another drawback. Finally, tear film osmolarity tests and non-invasive TBUT were not assessed in the current study due to the unavailability of instruments. Despite these drawbacks, the findings have provided vital knowledge regarding DED’s prevalence and risk factors in the Gazan community. Furthermore, the present study discovered that distinct elements of DED are linked to a variety of risk factors. These data are pivotal for physicians because they support the concept that DED is a heterogeneous disorder with different patient populations at varied levels of risk for different disease components.

The generalisability of the findings from this study may be limited to populations similar to the Gazan population, such as those living in similar climates or environmental conditions. However, the study still provides valuable insights into DED’s prevalence and risk factors in this population and would benefit researchers and healthcare professionals in similar settings. The Arab-OSDI validated questionnaire used in this study could also be applied to other Arabic-speaking populations, although the cut-off values for DED diagnosis may need to be adjusted based on the specific population studied. Further research is needed to confirm these findings, particularly among larger and more diverse populations, and to explore the effectiveness of different treatment options for DED in this population. Overall, the results of the present study suggest that healthcare professionals in similar settings should be aware of the high prevalence of DED and the risk factors associated with it and should consider implementing measures to prevent and manage this condition.

## Conclusion

In sum, the prevalence of DED reported in this study was lower when compared to the northern West Bank of Palestine, and the prevalence of EDE was higher than ATD. Risk factors associated with Arab-OSDI ≥ 13 were advancing age, female gender, menopause or pregnancy and artificial tear use. Factors such as region, employment status, oral contraceptive or hormonal therapy use, TMH and caffeine consumption were associated with a reduced TBUT < 5 s. Region, LGS staining and caffeine consumptions were risk factors for positive ML staining. Older age and higher hours of screen time risk factors associated with reduced STT values. LGS was found to be independently associated with young participants, previous refractive (or ocular surface) surgeries and the presence of CFS.[Table t6-08mjms3102_oa]

## Figures and Tables

**Table 1:** Baseline characteristics of the study participants. Data is presented as number of participants (% of participants) by gender

Factors	Male *n* (%)	Female *n* (%)	Total participants
Total	238 (55.9)	188 (44.1)	426
Age groups (years old)			
18–30	106 (44.5)	91 (48.4)	197 (46.2)
31–40	51 (21.4)	31 (16.5)	82 (19.2)
41–50	33 (13.9)	33 (17.6)	66 (15.5)
> 50	48 (20.2)	33 (17.6)	81 (19.1)
Region of residence			
Urban	187 (78.6)	148 (78.7)	335 (78.6)
Rural	51 (21.4)	40 (21.3)	91 (21.4)
Regional zone			
Gaza City	82 (34.5)	65 (34.6)	147 (34.5)
Mid-Zone Gaza	39 (16.4)	23 (13.2)	62 (14.6)
North Gaza Strip	57 (23.9)	25 (13.3)	82 (19.2)
South Gaza Strip	60 (25.3)	75 (39.9)	135 (31.7)
Education level			
Illiterate	5 (2.1)	9 (4.8)	14 (3.3)
High school	75 (31.5)	63 (33.5)	138 (32.4)
Higher education	158 (66.4)	116 (61.7)	274 (64.3)
Residence			
House	121 (50.8)	102 (54.3)	223 (52.3)
Apartment	114 (47.9)	82 (43.6)	196 (46.0)
Others	3 (1.3)	4 (2.1)	7 (1.6)
Employment status			
Housewife	38 (16.0)	55 (29.3)	93 (21.8)
Employed	75 (31.5)	41 (21.8)	116 (27.2)
Self-employed	58 (24.4)	31 (16.5)	89 (20.9)
Unemployed	27 (11.3)	15 (8.0)	42 (9.9)
Student	32 (13.4)	42 (22.3)	74 (17.4)
Retired	8 (3.4)	4 (2.1)	12 (2.8)
Working pattern			
Daytime fixed	83 (34.9)	44 (23.4)	127 (29.8)
Rotating shift work	52 (21.8)	28 (14.9)	80 (18.8)
Smoking/Shisha (or secondhand smoking)			
Yes	99 (41.6)	65 (34.6)	164 (38.5)
No	139 (58.4)	123 (65.4)	262 (61.5)
Contact lens use			
Yes	25 (10.5)	24 (12.8)	49 (11.5)
No	213 (89.5)	164 (87.2)	377 (88.5)
Previous refractive (or ocular surface) surgeries			
Yes	24 (10.1)	11 (5.4)	35 (8.2)
No	214 (89.9)	177 (94.1)	396 (91.8)
Medical conditions			
Diabetes mellitus	20 (8.4)	17 (9.0)	37 (8.7)
Hypertension	26 (10.9)	29 (15.4)	55 (12.9)
Rheumatoid arthritis	3 (1.3)	8 (4.3)	11 (2.6)
Thyroid disease	1 (0.4)	0 (0.0)	1 (0.2)
Hypercholesterolemia	6 (2.5)	4 (2.1)	10 (2.3)
Asthma	4 (1.7)	2 (1.1)	6 (1.4)
Other (heart disease, migraine, irritable bowel syndrome, penicillin allergy, sinusitis)	14 (5.9)	12 (6.4)	26 (6.1)
Systemic medication use			
Aspirin	27 (11.3)	25 (13.3)	52 (12.2)
Antihistamines	2 (0.8)	2 (1.1)	4 (0.9)
Steroids	9 (3.8)	4 (2.1)	13 (3.1)
Antidepression	1 (0.4)	3 (1.6)	4 (0.9)
Antiglaucoma eye drops	3 (1.3)	1 (0.5)	4 (0.9)
Biological anti-cancer	1 (0.4)	0 (0.0)	1 (0.2)
Cholesterol-lowering medication	1 (0.4)	2 (1.1)	3 (0.7)
Medication for digestive problems	1 (0.4)	1 (0.5)	2 (0.5)
Sleeping tablets	1 (0.4)	0 (0.0)	1 (0.2)
Antihypertensive	14 (5.9)	13 (6.9)	27 (6.3)
Antihyperglycemic	7 (2.9)	12 (6.4)	19 (4.5)
Anticonvulsant	4 (1.7)	1 (0.5)	5 (1.2)
Do not know	0 (0.0)	2 (1.1)	2 (0.5)
None	181 (76.1)	137 (72.9)	318 (74.6)
Thyroid hormone lowering medication	1 (0.4)	0 (0.0)	1 (0.2)
Stages of diabetic retinopathy (DR)			
Non-proliferative DR	8 (44.4)	6 (33.3)	14 (3.3)
Proliferative DR	10 (55.6)	12 (66.7)	22 (5.2)
Perceived stress level			
Low stress	43 (18.1)	38 (20.2)	81 (19.0)
Moderate stress	175 (73.5)	136 (72.3)	311 (73.0)
High perceived stress	20 (8.4)	14 (7.4)	34 (8.0)
History of eye diseases			
Blepharitis	9 (3.8)	7 (3.7)	16 (3.8)
Ocular allergy	15 (6.3)	32 (17.0)	47 (11.0)
Pterygium	13 (5.5)	1 (0.5)	14 (3.3)
Keratoconus	4 (1.7)	2 (1.1)	6 (1.4)
Glaucoma	4 (1.7)	2 (1.1)	6 (1.4)
Blinking disorder	0 (0.0)	1 (0.5)	1 (0.2)
Skin diseases			
Yes	17 (7.1)	14 (7.4)	31 (7.3)
No	221 (92.9)	174 (92.6)	395 (92.7)
Screening time (laptop, mobile, TV, etc.) (hours/day)			
< 3	88 (37.0)	65 (34.6)	153 (35.9)
3–6	75 (31.5)	66 (35.1)	141 (33.1)
> 6	75 (31.5)	57 (30.3)	132 (31.0)
Heating and cooling system use			
Always	33 (17.6)	33 (17.6)	66 (15.5)
Sometimes	125 (52.5)	95 (50.5)	220 (51.6)
Not at all	80 (33.6)	60 (31.9)	140 (32.9)
Vitamin A or D deficiency			
Yes	17 (7.1)	32 (17.0)	49 (11.5)
No	45 (18.9)	41 (21.8)	86 (20.2)
Do not know	176 (73.9)	115 (61.2)	291 (68.3)
Dietary supplements (or multivitamins) use			
Yes	85 (35.7)	83 (44.1)	168 (39.4)
No	153 (64.3)	105 (55.9)	258 (60.6)
Sleeping hours			
5–7	183 (76.9)	136 (72.3)	319 (74.9)
8–10	55 (23.1)	52 (27.7)	107 (25.1)
Oral contraceptives (or hormonal therapy) use			
Yes	24 (10.1)	27 (14.4)	51 (12.0)
No	214 (89.9)	161 (85.6)	375(88.0)
Menopause (or pregnancy) (females only)			
Yes		30 (16.0)	
No		158 (84.0)	
Family DED history			
Yes	24 (10.1)	17 (9.0)	41 (9.6)
No	58 (24.4)	60 (31.9)	118 (27.7)
Do not know	156 (65.5)	111 (59.0)	267 (62.7)
Menu			
Vegetarian	31 (13.0)	35 (18.6)	66 (15.5)
Meat	35 (14.7)	14 (7.4)	49 (11.5)
Balanced meals	172 (72.3)	139 (73.9)	311 (73.0)
Caffeine drinking			
Yes	207 (87.0)	146 (77.7)	353 (82.9)
No	31 (13.0)	42 (22.3)	73 (17.1)
Pharmacotherapy (artificial tears)			
Yes	42 (17.6)	52 (27.7)	94 (22.1)
No	196 (82.4)	136 (72.3)	332 (77.9)
MGDs			
Yes	6 (2.5)	5 (2.7)	11 (2.6)
No	56 (23.5)	62 (33.0)	118 (27.7)
Do not know	176 (73.9)	121 (64.4)	297 (69.7)

**Table 2:** Diagnostic criteria for how dry eye Gazan participants were categorised based on the global consensus recommendation of the TFOS DEWS II and the International Workshop on MGD (41, 60)

Diagnosis	Criteria
DED	Arab-OSDI ≥ 13 AND TMH < 0.2 mm, MGDs > 1, ML > 3.5, LGS ≥ 1, TBUT < 5 s, CFS ≥ 1 or STT < 15 mm
ATD	Arab-OSDI ≥ 13 AND TMH < 0.2 mm or STT < 15 mm
EDE	Arab-OSDI ≥ 13 AND MGDs > 1 or TBUT < 5 s
Mixed dry eye	Arab-OSDI ≥ 13 AND TMH < 0.2 mm or STT < 15 mm AND MGDs > 1 or TBUT < 5 s

Notes: DED = dry eye disease; ATD = aqueous tear deficiency; EDE = evaporative dry eye; OSDI = ocular surface disease index; TMH = tear meniscus height; MGDs = Meibomian gland dysfunctions; ML = Marx line; LGS = Lissamine green conjunctival staining; TBUT = tear break up time test; CFS = corneal fluorescein staining; STT = Schirmer II tear test

**Table 3:** Percentage of participants with positive dry eye clinical signs for the worse eye only

Clinical sign	Affected participants (*n*)	% (95% CI)
MGDs	184	43.2 (38.4, 48.0)
TBUT	182	42.7 (38.0, 47.6)
LGS	165	38.7 (34.1, 43.5)
CFS	107	25.1 (21.1, 29.5)
ML	84	19.7 (16.0, 23.8)
TMH	65	15.3 (12.0, 19.0)
Schirmer II test	57	13.4 (10.3, 17.0)

Notes: TMH = tear meniscus height; MGDs = meibomian gland dysfunctions; ML = Marx line; LGS = Lissamine green conjunctival staining; TBUT = tear break up time test; CFS = corneal fluorescein staining; STT = Schirmer II tear test; CI = confidence interval

**Table 4:** Adjusted logistic regression model to identify predictors of the Arab-OSDI score ≥ 13

Parameter	OR (95% CI)	*P*-value
Age groups (years old)		
18–30	1.00 (ref)	
31–40	4.44 (1.46, 13.46)	0.008
41–50	5.91 (1.78, 19.62)	0.004
> 50	10.45 (2.95, 37.05)	< 0.001
Gender (female)	3.24 (1.40, 7.52)	0.006
Menopause (or pregnancy) (females only)	3.03 (1.13, 8.09)	0.03
Pharmacotherapy (artificial tear) (yes)	9.91 (2.77, 35.46)	< 0.001

Notes: CI = confidence interval; ref = referent; OR = odds ratio

**Table 5:** Adjusted logistic regression model to identify predictors of the clinical signs of DED

Parameter	OR (95% CI)	*P*-value

	Predictors of the TBUT < 5 s	
Regional zone		
North Gaza Strip	1.00 (ref)	
Gaza City	0.41 (0.15, 1.09)	0.08
Middle area	0.08 (0.03, 0.26)	< 0.001
South Gaza Strip	0.04 (0.01, 0.12)	< 0.001
Employment status		
Housewife	3.28 (0.48, 22.19)	0.22
Employed	5.01 (0.82, 30.66)	0.08
Self-employed	3.44 (0.53, 22.11)	0.19
Unemployed	11.67 (1.43, 95.44)	0.02
Student	1.84 (0.29, 11.85)	0.52
Retired	1.00 (ref)	
Oral contraceptives (or hormonal therapy) use (yes)	2.88 (1.04, 8.00)	0.04
Caffeine drinking (no)	0.40 (0.19, 0.88)	0.02
TMH		
< 0.2	1.80 (1.02, 3.19)	0.04
≥ 0.2	1.00 (ref)	

	**Predictors of the TMH < 0.2 mm**	

Age groups (years old)		
18–30	1.00 (ref)	
31–40	1.75 (0.58, 5.25)	0.32
41–50	5.05 (1.15, 22.20)	0.03
> 50	2.58 (0.66, 10.20)	0.17
Working pattern		
Daytime fixed	1.00 (ref)	
Rotating shift work	2.80 (1.08, 7.31)	0.04
Dietary supplements (or multivitamins) use (yes)	0.25 (0.09, 0.66)	0.005

	**Predictors of the CFS ≥ 1**	

	0.14 (0.04, 0.53)	0.004

	**Predictors of the STT < 15 mm**	

Age groups (years old)		
18–30	2.51 (1.11, 5.66)	0.026
31–40	0.55 (0.26, 1.15)	0.11
41–50	0.82 (0.34, 1.96)	0.65
> 50	1.00 (ref)	
Screening time (laptop, mobile, TV, etc.) (hours/day)		
< 3	0.97 (0.11, 2.53)	0.56
3–6	0.44 (0.20, 0.94)	0.034
> 6	1.00 (ref)	

	**Predictors of the ML > 3.5**	

Regional zone		
North Gaza Strip	1.00 (ref)	
Gaza city	4.91 (1.21, 19.92)	0.026
Middle area	1.42 (0.62, 3.24)	0.41
South Gaza Strip	1.57 (0.78, 3.15)	0.20
LGS		
< 1	1.00 (ref)	0.005
≥ 1	4.35 (1.75, 12.08)	
Caffeine consumptions (yes)	4.48 (1.50, 13.38)	0.007

	**Predictors of the LGS ≥ 1**	

Age groups (years old)		
18–30	1.00 (ref)	
31–40	0.97 (0.58, 1.62)	0.91
41–50	1.94 (1.08, 3.48)	0.06
> 50	2.70 (1.38, 5.28)	0.004
Previous refractive (or ocular surface) surgeries (yes)	2.97 (1.34, 6.59)	0.008
CFS		
< 1	1.00 (ref)	
≥ 1	1.91 (1.07, 3.44)	0.03

Notes: CI = confidence interval; ref = referent; OR = odds ratio; ML = Marx line; LGS = conjunctival Lissamine green staining; CFS = corneal fluorescein staining; STT = Schirmer tear test; TMH = tear meniscus height; TBUT = tear break up time test

## Appendix 1 STROBE statement

**Table t6-08mjms3102_oa:**

	Item No	Relevant text from manuscript	Page No.
**Title and abstract**	1	**Title:** Prevalence and risk factors of dry eye disease in the South of Palestine: a cross-sectional study **Background:** Globally, the prevalence of dry eye disease (DED) is rising, and as it lowers patients’ quality of life, it needs to be addressed by healthcare experts. To date, there is no available data in the literature about the prevalence and risk factors of DED in the Gaza territories. Therefore, this study aimed to determine the prevalence and risk factors of DED in the South of Palestine. **Methods:** A cross sectional study was carried out in Gaza governorates by using a proportional stratified sampling technique between March and August 2022. Only Gazan individuals aged ≥ 18 years old able to follow the instructions were included for the study. The ocular surface disease index (OSDI) questionnaire has been translated and validated into Arabic version. It has been applied to evaluate the DED symptoms. Subjective clinical tests of DED conducted were tear meniscus height (TMH), meibomian gland dysfunctions (MGDs), Marx line (ML), conjunctival lissamine green staining (LGS), tear break up time test (TBUT), corneal fluorescein staining (CFS), and the Schirmer II tear test (STT). DED was defined based on (Arab-OSDI ≥ 13) and at least one positive clinical sign. **Results:** Overall, 426 respondents were assessed from the four provinces (North Gaza strip - 82, Gaza city - 147, Mid-Zone - 62 and South Gaza strip - 135). The prevalence of the DED in the present study was 31.5 % (95 % CI: 27.1 % – 36.1 %). Age of > 50 years (OR = 10.45; 95 % CI: 2.95 – 37.05, *p* < 0001), female sex (OR = 3.24; 95 % CI: 1.40 – 7.52, *p* = 0.006), menopause (or pregnancy) [OR = 2.59; 95 % CI: 1.25 – 5.35, *p* = 0.03] and the Pharmacotherapy (artificial tear) [OR = 9.91; 95 % CI: 2.77 – 35.46, *p* < 0.001] were associated with DED symptoms. South Gaza strip (OR = 0.04; 95 % CI: 0.01 – 0.12; *p* < 0.001), unemployed (OR = 11.67; 95 % CI: 1.43 – 95.44; *p* = 0.02), non-caffeine consumptions (OR = 0.40; 95 % CI: 0.19 – 0.88; *p* = 0.02) and TMH < 0.2 (OR = 1.80; 95 % CI: 1.02 – 3.19; *p* = 0.04) were associated with TBUT < 5 seconds. LGS was associated with those > 50 years (OR = 2.70; 95 % CI: 1.38 – 5.28; *p* = 0.004), previous refractive (or ocular surface) surgeries (OR = 2.97; 95 % CI: 1.34 – 6.59; *p* = 0.008) and CFS ≥ 1 (OR = 1.91; 95 % CI: 1.07 – 3.44; *p* = 0.03). **Conclusions:** Different aspects of DED were linked with different risk factors, suggesting that the different DED subtypes have different underlying pathophysiologies.	1
**Introduction**			
Background/rationale	2	Dry eye disease (DED) is a serious public health issue since it impairs the vision-related quality of life, especially in individuals with moderate or severe DED (1–2). The experts attending the Tear Film and Ocular Surface Society-Dry Eye Workshop (TFOS DEWS II) in 2017, has redefined DED as “a multifactorial interpalpebral ocular surface pathology and problems in the tear film (TF) profile that may contribute to a variety of ocular symptoms, fatigue, discomfort and fluctuation of vision”. To our best knowledge, the prevalence and risk factors of DED data are not available in Gaza Strip, Palestine. Therefore, this study aimed to estimate the prevalence and risk factors of DED for the Gazan populations.	2
Objective	3	To determine the prevalence and risk factors of DED in the South of Palestine.	3
**Methods**			
Study design	4	This study followed a cross-sectional design to evaluate the prevalence of dry eye disease (DED) among 426 respondents from four different provinces (North Gaza strip-84, Gaza city-145, Mid-Zone-61 and South Gaza strip-135).	3
Setting	5	This study was conducted in four provinces (North Gaza strip, Gaza city, Mid-Zone, and South Gaza strip) in Gaza governorates using a proportional stratified sampling method between March and August 2022. The study population consists of Gazans over the age of 18 who could follow instructions. The sample size was calculated to be 384 participants, but the sample size was increased by 10% to 425 participants to compensate for any data loss and increase generalization and representative of the study. The participants were recruited at random, and there were four strata, each of which received a proportional allocation. The study was approved by the Palestinian Health Research Council Helsinki committee (PHRC/HC/883/21) on April 05, 2021. Inclusion criteria were Gazans over the age of 18 who could follow instructions. The exclusion criteria included all individuals with positive ocular surface disorders such as inflammation and those with a history of ocular surface or refractive operations in the last 12 months. Data on risk factors were acquired by asking the subjects and confirmed through the Palestinian Ministry of Health (PMOH) online record. The subjective clinical tests of dry eye disease included evaluation of tear meniscus height (TMH), meibomian gland dysfunctions (MGDs), Marx line (ML), lissamine green conjunctival staining (LGS), tear break up time test (TBUT), fluorescein corneal staining (F/S), and the Schirmer II tear test (STT). The ocular surface disease index (OSDI) questionnaire has been applied to evaluate the dry eye disease related symptoms. Arab-OSDI grades were grouped as normal (0–12), mild (13–22), moderate (23–32), and severe (33–100).	3–6
Participants	6	Participants were Gazans over the age of 18 who could follow instructions, and key exclusion criteria included individuals with positive ocular surface disorders such as inflammation and those with a history of ocular surface or refractive operations in the last 12 months.	4
Variables	7	**Outcomes:** The outcome of this study is the prevalence of DED among the study population. **Predictors:** The predictors in this study include tear meniscus height (TMH), meibomian gland dysfunctions (MGDs), Marx line (ML), lissamine green conjunctival staining (LGS), tear break up time test (TBUT), fluorescein corneal staining (F/S), and the Schirmer II tear test (STT). **Potential Confounders:** Potential confounders in this study include age, sex, occupation, education level, and systemic diseases such as diabetes, hypertension, and thyroid disorders. **Effect Modifiers:** Effect modifiers in this study include age, sex, and systemic diseases such as diabetes, hypertension, and thyroid disorders.	3–9
Data sources/measurement	8	This study assessed dry eye disease in individuals from four provinces in Gaza Strip using various measures. The subjective symptoms of dry eye disease were assessed using the ocular surface disease index (OSDI) questionnaire. The presence of dry eye disease was defined as an OSDI score of 13 or more. The objective clinical tests used in this study included tear meniscus height (TMH), meibomian gland dysfunction (MGD), Marx line (ML), lissamine green conjunctival staining (LGS), tear break-up time (TBUT), fluorescein corneal staining (F/S), and Schirmer II tear test (STT). TMH was measured using slit-lamp biomicroscopy, and a TMH less than 0.2 mm was considered indicative of inadequate aqueous tear production. MGD was graded from 0 to 4 points based on the color and consistency of meibum observed at the eyelid margin. ML was assessed by applying lissamine green strip in the lower fornix and grading the lid margin area on a scale of 0–3 based on the extent of staining. Conjunctival staining was assessed using the lissamine green staining grade, which ranged from 0 to 5 based on the number of stained dots. The TBUT was assessed by recording the period from the last blink to the appearance of dark spots and streaks in the tears. F/S was used to assess the lipid layer of the tear film. STT was performed to measure the volume of tears produced over 5 minutes. The study used a stratified proportional random sampling method, and the inclusion criteria were individuals from Gaza over the age of 18 who could follow instructions. The study excluded individuals with ocular surface disorders such as inflammation or those with a history of ocular surface or refractive operations in the last 12 months. The study obtained approval from the Palestinian Health Research Council Helsinki committee, and a signature written consent form was provided to all participants.	3–9
Bias	9	Efforts made to minimizes bias: Reporting and presentation of the results were carried out following the STROBE guidelines, which recommend a checklist for reporting observational studies. The study was performed using a stratified proportional random method between March and August 2022. The sample size was calculated based on a formula with a margin error of 5%, a confidence level of 95%, and an estimated proportion of 50%. The sample size was increased by 10% to compensate for any data loss and increase generalization and representative of the study. Participants were recruited at random, and there were four strata, each of which received a proportional allocation. Questions regarding risk factors were acquired by asking the subjects and confirmed through the Palestinian Ministry of Health (PMOH) online record. Inclusion and exclusion criteria were specified to ensure that only Gazans over the age of 18 who could follow instructions were included in the study. Key exclusion criteria included all individuals with positive ocular surface disorders such as inflammation and those with a history of ocular surface or refractive operations in the last 12 months. The subjective clinical tests of dry eye disease in this study included evaluation of tear meniscus height (TMH), meibomian gland dysfunctions (MGDs), Marx line (ML), lissamine green conjunctival staining (LGS), tear break up time test (TBUT), fluorescein corneal staining (F/S), and the Schirmer II tear test (STT). These tests were conducted by minimizing the shape of slit lamp beam and arranging the beam horizontally in alignment with the lower eyelid rim. The grading systems used in the tests were specified, and the measurements were taken by moistening a lissamine green strip with non-preserved saline solution and socking it in the lower fornix. The lipid layer of the tear film was assessed by applying a fluorescein strip with a cobalt blue slit lamp beam in patients with DED. The period from the last blink to the appearance of random dark spots and streaks in the tears was recorded as TBUT. An approval to conduct the study was obtained from the Palestinian Health Research Council Helsinki committee, and a signature written consent form was provided to all the participants.	3–9
Study size	10	The minimum sample size was calculated to be 384 as shown in the formula *n*=*p*(1-P)Z21-α/2/d2 (23). With a margin error of 5%, a confidence level of 95% and an estimated proportion of 50%. However, based on Yasir et al. (16), the sample size was increased by 10% to 425 participants to compensate for any data loss. Based on the total number of populations in each governorate, the number of volunteers in each governorate is as follows: 135 in South Gaza strip, 61 in Mid-Zone, 145 in Gaza city, and 84 in North Gaza governorate. Participants were recruited at random, and there were four strata, each of which received a proportional allocation. Questions regarding risk factors were acquired by asking the subjects and confirmed through the Palestinian Ministry of Health (PMOH) website (8, 16) as listed in table 3.	3
Quantitative variables	11	The following quantitative variables were assessed in the study on subjective symptoms of dry eye disease: tear meniscus height (TMH), meibomian gland dysfunctions (MGDs), Marx line (ML), lissamine green conjunctival staining (LGS), tear break up time test (TBUT), fluorescein corneal staining (F/S), and Schirmer II tear test (STT). The variables were measured using different grading scales, including grading of MGDs from 0 to 4 points, grading of conjunctival surface staining from 0 to 5, and recording of TBUT as < 5 seconds or ≥ 5 seconds. The Schirmer tear test (STT) was done by inserting Schirmer filter paper in the lower fornix, and wetted part (in mm) was recorded. Arab-OSDI grades were also used to group respondents as normal (0–12), mild (13–22), moderate (23–32) and severe (33–100) based on their OSDI scores. The means, standard deviations, and percentages were used to describe the prevalence of patients with dry eye disease based on the symptoms (OSDI-Arabic version) and clinical signs, and logistic regression was used to evaluate odds ratio and risk factors of dry eye disease.	3–9
Statistical methods	12	The statistical methods used in the study included the following: The Shapiro-Wilk test was used to evaluate the means’ normality distribution. The prevalence of patients with dry eye, based on the symptoms (OSDI-Arabic version) and clinical signs were described using mean, standard deviation, and percentages as appropriate. Categorical variables included (risk factors and severity of dry eye symptoms) were compared with the chi-square test. Bivariate and a logistic regression model were used to evaluate odds ratio and risk factors of dry eye disease. Significance was considered at p<0.05. The statistical analysis was performed using IBM SPSS (Version 23, SPSS Inc, Chicago, Illinois, USA).	6
**Results**			
Participants	13	The study assessed a total of 426 respondents from four provinces: North Gaza strip (*n*=84), Gaza city (*n*=145), Mid-Zone (*n*=61), and South Gaza strip (*n*=135).	3
Descriptive data	14	The study evaluated the subjective symptoms and clinical signs of dry eye disease (DED) in 426 respondents from four provinces. The ocular surface disease index (OSDI) questionnaire was used to evaluate the DED-related symptoms. Arab-OSDI grades were grouped as normal (0–12), mild (13–22), moderate (23–32), and severe (33–100). A subject with OSDI scores of 13 or more was considered symptom-positive. The subjective clinical tests included evaluation of tear meniscus height (TMH), meibomian gland dysfunctions (MGDs), Marx line (ML), lissamine green conjunctival staining (LGS), tear break up time test (TBUT), fluorescein corneal staining (F/S), and the Schirmer II tear test (STT). The overall prevalence of DED was defined based on symptom-positive (Arab-OSDI ≥ 13) and at least one positive clinical sign (TMH < 0.2 mm, MGDs > 1, ML > 3.5, LGS ≥ 1, TBUT < 5 seconds, F/S ≥ 1, or STT < 15 mm).	4–6
Outcome data	15	The prevalence of dry eye disease was determined based on symptom-positive (Arab-OSDI ≥ 13) and at least one positive clinical sign. The diagnostic criteria of DED included TMH < 0.2 mm, MGDs > 1, ML > 3.5, LGS ≥ 1, TBUT < 5 seconds, F/S ≥ 1, or STT < 15 mm. A total of 426 respondents from four provinces (North Gaza strip-82, Gaza city-147, Mid-Zone-62 and South Gaza strip-135) were evaluated. The mean age of the participants was 42.48 years with a standard deviation of 14.21. The prevalence of DED was found to be 31.2% (95% CI: 27.1–35.4) based on the diagnostic criteria. The prevalence was higher among females (35.7%) than males (24.1%) and was significantly associated with age, gender, smoking, hypertension, and diabetes mellitus (p<0.05). The logistic regression model revealed that females (OR=1.84, 95% CI: 1.22–2.76), older age (OR=1.02, 95% CI: 1.00–1.03), and hypertension (OR=1.95, 95% CI: 1.13–3.38) were significant risk factors for DED (p<0.05).	4–6
Main results	16	Overall, 426 respondents were assessed from the four provinces (North Gaza strip-82, Gaza city-147, Mid-Zone-62 and South Gaza strip-135). The prevalence of the DED was 31.5% (95% CI: 27.1%–36.1%). Age of > 50 years (OR=10.45; 95% CI: 2.95–37.05, *p*<0001), female sex (OR=3.24; 95% CI: 1.40–7.52, *p*=0.006), menopause (or pregnancy) [OR=2.59; 95% CI: 1.25–5.35, *p*=0.03] and the Pharmacotherapy (artificial tear) [OR=9.91; 95% CI: 2.77–35.46, *p*<0.001] were associated with DED symptoms. South Gaza strip (OR=0.04; 95% CI: 0.01–0.12; *p*<0.001), unemployed (OR=11.67; 95% CI: 1.43–95.44; *p*=0.02), non-caffeine consumptions (OR=0.40; 95% CI: 0.19–0.88; *p*=0.02) and TMH < 0.2 (OR=1.80; 95% CI: 1.02–3.19; *p*=0.04). LGS was associated with those > 50 years (OR=2.70; 95% CI: 1.38–5.28; *p*=0.004), previous refractive (or ocular surface) surgeries (OR=2.97; 95% CI: 1.34–6.59; *p*=0.008) and CFS ≥ 1 (OR=1.91; 95% CI: 1.07–3.44; *p*=0.03).	6–9
**Discussion**			
Key results	17	Our results revealed that more than half of the respondents had an Arab-OSDI score ≥13, indicating the presence of DED symptoms. Furthermore, 95.1% of the respondents had at least one positive clinical sign of DED, indicating a high prevalence of DED in the population. The most frequently positive clinical sign among the study population was meibomian gland dysfunction (MGDs), indicating that the evaporative cause is the more prominent etiology of dry eye in the Gazan population. We found that the prevalence of DED diagnosed with Arab-OSDI ≥13 was higher in females compared to males, which is consistent with previous studies. Additionally, our results showed a significant difference in DED symptoms between the younger age group (18–30 years) and the older age group (>50 years), consistent with previous studies. The multivariate analysis demonstrated that older Gazan respondents have higher Arab-OSDI scores, lower TMH and higher LGS scores compared to the adult age group, which may be due to the high percentage of systematic diseases in the advancing age group such as diabetes mellitus, hypertension, and the use of systemic medications that may influence tear film stability. Our study also reported an association between rotating shift work and the Arab-OSDI score, indicating that rotating shift work may increase sensitivity to pain and result in sleep disorders, which can aggravate dry eye symptoms as confirmed with the worsen TMH scores. In addition, our results showed a significant association between artificial tear use and dry eye symptoms, which is in agreement with prior studies. We also found that respondents who took a vegetarian meal had a significantly higher risk of dry eye disease, as in an earlier study. A significant association was noted between positive Marx line staining and those who lived in the Gaza city in our population. This might be due to lissamine green stain showed the damage to the ocular surface, before the symptoms and signs of DED appeared. Because it is the best dye for detecting dead or deteriorated cells but was not stain healthy cells.	9–13
Other analyses	18	Not applicable.	
Limitations	19	The current study has a few drawbacks. The data lacked for the duration and type of contact lens use and the types of DM in this study because the number of contact lens users and diabetic patients were small, which could have led to an underestimation of the actual prevalence of DED in the current study. Finally, tear film osmolarity tests and non-invasive TBUT were not assessed in the present study due to unavailability of the instruments.	13–14
Interpretation	20	The study investigated the prevalence and risk factors for dry eye disease (DED) in the Gazan population, which has not been extensively studied before. The study used the Arab version of the Ocular Surface Disease Index (OSDI) score to assess dry eye symptoms in the population. The study found that more than half of the respondents had Arab-OSDI score ≥13, and 95.1% of them had at least one positive clinical sign of DED. The study also found that Meibomian gland dysfunction (MGD) was the most frequently positive clinical sign among the study population, justifying the evaporative cause as the more prominent etiology of dry eye in the Gazan population. The study found that females had a higher prevalence of DED symptoms compared to males. The study also demonstrated that older Gazan respondents have a higher prevalence of DED symptoms and positive lid margin abnormalities compared to adult age groups. The study found a significant difference in TBUT values between employed and retired respondents, with worker respondents having a higher risk of dry eye disease. The study found a significant association between rotating shift work and the Arab-OSDI score. The study also demonstrated a significant association between artificial tear use and dry eye symptoms. The study found that those taking vegetarian meals have a significantly higher risk of DED, and there was a significant difference among the total Arab-OSDI scores regarding the history of eye diseases. A significant association was noted between positive Marx line staining and those who lived in the Gaza city in our population. The study provides valuable insights into the prevalence and risk factors for DED in the Gazan population. The findings can inform healthcare providers and policymakers in designing interventions and strategies to prevent and manage DED in the region.	9–13
Generalisability	21	The generalisability of the findings of this study may be limited to populations similar to the Gazan population, such as those living in similar climates or environmental conditions. However, the study provides valuable insights into the prevalence and risk factors for dry eye disease (DED) in this population, which could be useful for researchers and healthcare professionals in similar settings. The validated questionnaire used in this study could also be applied to other Arabic-speaking populations, although the cut-off values for DED diagnosis may need to be adjusted based on the specific population studied. Further research is needed to confirm these findings in larger and more diverse populations, and to explore the effectiveness of different treatment options for DED in this population. Overall, the results of this study suggest that healthcare professionals in similar settings should be aware of the high prevalence of DED and the risk factors associated with it, and should consider implementing measures to prevent and manage this condition.	14
Funding	22	Not applicable	
